# Visual Disabilities in Children Including Childhood Blindness

**DOI:** 10.4103/0974-9233.51988

**Published:** 2008

**Authors:** Rajiv Khandekar

**Affiliations:** From the Department for Control of Non Communicable diseases, Director General of Health Affairs, Ministry of Health, Oman

**Keywords:** visual disabilities, childhood blindness, VISION 2020, congenital cataract, retinopathy of prematurity, low vision care

## Abstract

We should address visual disabilities in children instead of only the childhood blindness. Diseases related to nutritional, communicable diseases should be addressed through strategies for achieving ‘Millennium Development Goals’. Facilities in African countries and countries with populations like India and China must be strengthened to address curable/preventable visual disabilities in children. Even though all efforts are done to strengthen, we will have 0.93 million blind children by 2020. Role of family physicians and paediatricians in trans-disciplinary approach to address visual disabilities in children is very crucial. If rational distribution of skilled human resource is not planned visual disabilities will not reduce effectively. Rehabilitation of visually disabled children should be integral part of addressing childhood blindness. All stakeholders including parents of children with visual disabilities should work together to achieve the goals.

Childhood blindness refers to a group of diseases and conditions occurring in childhood or early adolescence (<16 years of age), which, if left untreated, result in blindness or severe visual impairment that are likely to be untreatable later in life.^1^ Due to enormous loss of Disability Adjusted Life Years (DALYs), childhood blindness is estimated to be the second leading cause of the burden due to blindness.^2^ The global cost of blindness with the onset in childhood in terms of lost capacity of earning has been estimated to be between US$ 6,000 and $27,000 million.^3^ Visual disabilities in children are more complex compared to those in adults. Without visual stimulus, the child's overall development suffers. In addition to the disabled child, his/her family and the society at large are also negatively affected. Therefore, the World Health Organization and its partners in their consorted efforts to eliminate avoidable blindness ‘VISION 2020’ The Right to the Sight' included childhood blindness also.

While reviewing the global situation of childhood blindness, it is essential to project the magnitude and debate the rationale of grouping different types of visual disabilities.

## Magnitude of Blindness in Children and Projections:

**Figure 1:**
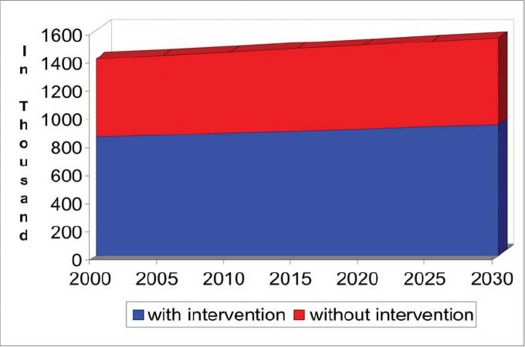
Projections of bilateral blind (vision <3/60) among <16 years old children.

The prevalence of childhood blindness was 0.3 per 1,000 children in industrialised countries and 1.2 per 1,000 children in the developing countries in the year 2000. Accordingly, it was estimated that there were nearly 1.4 million blind children in the world. Each year, an additional 50,000 children become blind and are added to this pool.^4^ We made an attempt to project the magnitude of blind children up to the year 2030 and for this evidence based information and few assumptions were used (Figure 1). It is a well known fact that 40% of childhood blindness is due to preventable/curable causes.^5^ While projecting the magnitude by time in a chronic condition, childhood mortality rate should be accounted for. A study in UK suggested that 10% of children with severe visual impairment and blindness die within the first year of detection of their blindness because many them have other potentially life threatening conditions.^6^ In another study in Sweden, 13% of blind children died due to other systemic conditions.^7^ Accordingly, world is likely to have 1.6 million blind children if no additional interventions are carried out. If all curable or avoidable causes are addressed, there would still be 0.93 million children with blindness. We have assumed that in next 25 years, no new intervention modalities shall be available to treat today's unavoidable causes (80% of childhood blindness) and we hope that that is not true. Considerable progress has been made in the field of genetics to locate disease related genes for congenital cataract, retinal diseases and conditions linked to cortical blindness.^8^,^9^ Scientists may soon find solutions to treat these conditions.

## What Definition of Blindness Should Be Used?

Rahi et al studied the epidemiology of visual impairment and blindness in childhood in 2005 and they highlighted the importance of uncorrected refractive error.^10^ Unfortunately, current WHO definition of “blindness” does not include uncorrected refractive error. It is a major problem in children especially in Asian countries. Many children in these countries have limited access to refractive services. In Arab countries access to visual aids is granted, but frequently not wanted for cultural reasons. Without visual aids, these children remain functionally blind and are unable to perform activities for daily living. Hence a revision of definition was proposed to include vision as presented rather than best-corrected vision while defining blindness. If we use the definition of blindness as vision <3/60 ‘presenting’ instead of ‘best corrected’, uncorrected refractive error would contribute 15% of the global blindness in children. WHO has recommended a revision of the estimates of blindness to include uncorrected refractive error.^11^

In industrialised countries and countries with rapidly developing economies like India, blindness is defined as vision <6/60 with best possible correction and corresponding restriction in field of vision. This is different from WHO recommended definition of blind. In view of high precision visual needs for daily activities, working on computer, entertainment through television, it is high time the definition of blindness especially in children is revised. If we make it stringent the magnitude of blindness in children will definitely increase. A population-based survey in South India had defined blindness as vision <6/60 as presented. On its basis the authors projected that India could have as many as 68,000 blind children.^12^ Global projections on basis of this definition should be carried out by 2010.

If a person with unilateral blindness has progressive eye disease or fellow eye is affected due to ocular trauma, he/she would become visually disabled in both eyes. Therefore unilateral blindness is included in calculation of disability percentage for compensation. Magnitude and epidemiology of unilateral blind children therefore would be of interest to the health planners. To our knowledge, data on the prevalence of unilateral blindness in children is scanty. A community based study of 6,610 children of <16 years of age in Oman suggested that the prevalence of unilateral blindness was 9 per 10,000 children. In the same age group the rate of bilateral blindness was 7 per 10,000.^13^ If visual disability due to unilateral blindness is added in estimating the burden of visual impairment, it is likely that global estimates visual impairment in children will double.

Many diseases causing visual disabilities in children show epidemiological transition. Nutrition and infection related causes mainly lead to corneal blindness. The citizens in countries facing wars and civil conflicts suffer from poverty and poor hygiene and Iraq has recently reported more blind children.^14^ Diabetes is a major challenge in countries with evolving economies and children with juvenile diabetes would suffer from visual impairment and would need periodic eye care.^15^ Improved health services will result in better survival of children with visual impairment and multi-system diseases. Thus in coming years their numbers might increase. However infants with severe birth defects become less as therapeutic abortions are more acceptable and hence the number of children with cerebral visual impairment is likely to decrease.^16^,^17^ In short, risk factors of childhood blindness in the next twenty years are likely to change, requiring periodic review of the world data on childhood blindness for proper planning.

## Causes of Childhood Blindness:

Childhood blindness could be grouped according to the anatomical structure affected or by the principal cause of visual disability.^18^ Both are important while evaluating the impact of public health initiatives. Blindness (involving the cornea) due to complications of measles, Vitamin A deficiency, ophthalmia neonatorum and harmful traditional eye medicines was common in the past in countries with poor economies. Uncorrected refractive error is also a major contributor to the visual disability that could be managed at primary health care level.

Congenital cataract and Retinopathy of Prematurity (ROP) affect visual functions in very early ages and they can be prevented or treated. Modalities to prevent, diagnose and manage these conditions are available.^19^,^20^ Visual prognosis after cataract surgery in young children has improved considerably. But, congenital and infantile cataracts are still responsible for 10% of global childhood blindness and the leading cause of blindness in many countries of Africa. For children with bilateral dense cataract, urgent surgery is recommended to avoid dense amblyopia. In eyes with congenital and developmental cataract that are associated with microphthalmos, microcornea, coloboma, as part of syndrome or if there is unilateral cataract, the visual outcomes are not very promising following their management.^21^,^22^ Risk of delayed complications like glaucoma, even after successful cataract surgeries in children often compromises the visual outcomes. Hence these children should be followed for a long time. Proper counselling of parents to ensure better compliance and follow up and setting up effective mechanisms to follow these operated children is crucial.

ROP is the fifth leading cause of childhood blindness globally. But fortunately, at risk infants who are screened and treated for ROP have better functional as well as structural results. The prevalence of aggressive ROP is linked to resuscitating premature babies of less than 1.5 kilo gram.^23^ The national programs should raise awareness among neonatologists and paediatricians for eye screening of ‘newborns at risk’ and create facilities to manage ROP cases. In view of the known substantial neuro-developmental retardation for life, ethical issues to interrupt the natural course of these pregnancies and outcomes should be weighed and proactive interventions should be reconsidered.

In many industrialised countries, diseases of the retina and optic nerve are now the leading causes of childhood blindness. As limited remedial measures are available to manage them, affected children will need rehabilitation. Further research on both to manage and rehabilitate these children even in developing countries should be encouraged.

Cortical visual impairment has become the major cause of visual impairment. The causes include perinatal hypoxia, hydrocephalus, meningitis, birth trauma, drowning episodes and periventricular leucomalacia. Intervention and educational strategies to rehabilitate visual function of such children have limited success but attempts will help the child, parents, teachers and physician.^24^

## Public Health Approach:

International organizations encourage and assist member countries to plan program approach to address childhood blindness. Their recommendations could be described as follows:

### Objectives:

The global aim is to reduce the prevalence of childhood blindness from 0.75:1000 to 0.4:1000 by the year 2020.

### Primary Prevention:

International organizations and national health programs should address all preventable causes of childhood blindness through primary prevention. They include universal immunization against measles and rubella and preventing and treating Vitamin A deficiencies. Most countries achieved high coverage of immunization and vitamin A supplementation initiatives.^18^ They could be implemented through child health care programs as it will also help in reaching the 4^th^ Millennium Developmental Goal.^25^ The risk of transmitting infection to the eyes of the foetus during intrauterine life and at the time of birth could be minimized by proper antenatal care and supervised/hospital deliveries. Early detection of birth defects by prenatal screening, proper counselling could also reduce childhood blindness associated with systemic conditions. Educational materials and counselling of parents are crucial. In Arab countries the population wearing a “visible prosthesis” is not very well accepted. Health promotion should address this issue. Establishing primary eye care and referral systems could prevent serious complications of common eye diseases in children.

**Status of Childhood Blindness in Next 5 to 10 Years:**

- Cataract secondary to rubella, measles and malnourishment will reduce and thus children with visual disabilitydue to them will decline.
- Visual disabilities due to uncorrected refractive error in children would be addressed well in developing countries.
- More ophthalmologists will be trained in paediatric ophthalmology but many of them will continue serving inurban areas of developing countries.
- Childhood blindness might get less attention compared to the other priority eye diseases included in the diseasecontrol strategy of ‘VISION 2020.’ (Senile cataract, Trachoma & Onchocerciasis)
- Rehabilitation of visually challenged children will need more attention as it is given to its prevention andmanagement.
- Evidence based health information would help in better planning of public health approach and rehabilitativeservices for different visual disabilities.

### Screening of Children with High Risk of Childhood Visual Disabilities:

Paediatric nurses, medical officers and paediatricians trained in eye screening could detect small or large eyeballs, nystagmus, strabismus, “white pupils” and birth defects like coloboma and aniridia. Proactive screening campaigns in institutions like blind schools, special education schools and preschool screening in kindergarten have been tried and are found cost effective.^26^ Such children should be reviewed at the earliest. Expectant mothers in communities with high consanguinity or with a family history of congenital anomalies of eyes should be closely monitored during pregnancy. Non-interventional screening procedures and genetic tests could identify gross eye anomalies.

### Strengthening Secondary/Tertiary Eye Care Units:

Examining eyes of very young children is challenging and not always possible for general ophthalmologists. In developing countries, skilled paediatric ophthalmologists and well-equipped subspecialty units are not easily accessible. General ophthalmologists with good diagnostic skills and effective equipment to note red reflex and refraction would be helpful. Ophthalmologists should be trained in detecting childhood related visual disabilities and they should promote long-term and frequent follow-ups. Orienting them about the significance of visual function would enable them to guide parents for rehabilitation of their children. An expert group was recently convened on the topic of childhood cataract, and guidelines are being developed.^27^ They were also described by Fan et al.^28^ Similar exercise for standardizing screening and managing ROP is also described.^29^ These protocols will assist ophthalmologists in improving their skills.

Although human resource development and technology at low cost are part of strengthening secondary and tertiary eye care, special attention should be given to apply these strategies.

### Human Resource Development:

It is recommended to have one paediatric eye centre with qualified paediatric ophthalmologist for 5 million population. The density of pediatric ophthalmologists was 1:4.5 million in South Africa in 2005, in India 0.63 per million in 2008.^30^,^31^ Resources to manage infantile and congenital cataract in developing countries are sparse and should be generated.^21^ Each center requires pediatric refractionists, orthoptists, low vision care technicians, paediatric counsellors and vocational trainers for providing practical training to visually disabled children. Community based management by ‘vision guardians’ can help to link tertiary services to the community. The training of health personnel of primary health care centres in basic eye examination, first aid and education of parents is needed. Such training programs could be provided by staff of tertiary level eye care centres. In African continent, cataract surgeons are trained for performing adult cataract surgeries but risk of complications in children with cataract and glaucoma are high and hence, they should manage cataract and glaucoma in children after getting special training in this field.

## Technologies for Management of Childhood Disease

Diagnosing eye ailments in young children is very challenging. Often prompt treatment is required but counselling parents of children with guarded prognosis is difficult. Proper diagnostic tools especially with digital and laser technologies have helped. But it is often difficult to keep pace with the rapid developments. Newer modalities are costly, not easy to procure and difficult to maintain. Scientifically conducted efficiency studies could guide clinicians and decision makers to procure such expensive but useful equipment. Operating on an infant requires expert anaesthetists and a well-equipped operation theatre. These children will need care and monitoring for the rest of their childhood and therefore computerized ‘case records’ would be an asset. Telemedicine could bring a child in need nearer to the expert situated at the other end of the world. Diagnostics and postoperative monitoring in remote areas could be supported after review of images by experts.

### Rehabilitation:

Although ophthalmic personnel do not offer rehabilitative services, they can motivate parents to avail them. Teachers and health personnel should encourage the child to use available residual vision. Such rehabilitation will need a trans-disciplinary approach involving a team comprising of an ophthalmologist, a paediatrician, parents, an optometrist, orthoptist, audiologist, a psychologist, psychiatrist, vision therapist, sociologist, and teachers. The goal is to integrate the child as soon as possible in a regular education system and not isolating him/her in institutions for children with special needs.

### Health Information and Research:

Many publications describe children in blind schools but few provide community-based information on childhood blindness. Large sample required for such studies, involving highly skilled manpower and high cost are main reasons for limited information. The impact of linking different health programs to address childhood blindness is another fertile area for research. 'Genetic epidemiology and genetic engineering' are the areas for future developments.

## Childhood Blindness Groups:

In developed countries, parents of affected children have created advocacy and self-help groups (e.g. Down Syndrome society, Autism group, Cerebral Palsy group, etc). Parents also unite to share their views, give confidence and comfort to the needy, fight for the rights of their children with special needs and even contribute in advocating policies for children with visual disability. Similar models may be adopted in other countries and can assist such groups partners in combating childhood visual disabilities.

### Perspective of the Blind:

One of the stakeholders of ‘VISION 2020’ is Blind Union. Members can contribute in social marketing of childhood blindness and formulating client friendly policies. Knowledge about rehabilitative opportunities is often not available with therapeutic caregivers. Therefore counselling by visually challenged and demonstrating their own examples will help parents to understand the situation, agree for timely interventions, cope with the stress and face newer challenges.

## Points to Ponder

Even though the focus is on diseases that cause visual disabilities, the indicators to monitor the progress of childhood blindness mainly focus on bilateral blindness. Unilateral blind, amblyopia, low vision and uncorrected refractive error might not be getting the attention that they deserve in many national health programs.

In many schools for the blind, nearly 40% children have residual vision. They are misfit in regular academic schools, but do not need to study Braille language. The approach to train those with absolute blind and children with residual vision separately should be explained to authorities properly.

Ophthalmologists focus mainly on operating cataracts as it is rewarding both financially as well as for satisfying the patient's visual needs. International organizations will focus on trachoma and onchocerciasis as they are public health problems in few countries and that too in limited areas. In this situation, childhood blindness in four priority diseases has the risk of being side-lined.

## Conclusions

‘Visual disabilities in children’ including the ‘Childhood Blindness’ should be addressed through a comprehensive program approach. In coming years should focus on integrated program approaches in countries of the African continent,^27^ India and China (with large populations) if significant reductions of curable/preventable childhood blindness are to be achieved.

We should rename priority eye disease ‘childhood blindness’ as ‘Visual disabilities in children’ in the ‘VISION 2020’ initiative. This will highlight importance of addressing unilateral blindness, low vision, amblyopia and uncorrected refractive errors. Visual disabilities in children due to nutritional deficiencies, measles, rubella and uncorrected refractive errors should be managed if countries attempt to reach the ‘Millennium Development Goals (MDGs)’. Even if we address all avoidable blindness we will be left with around 1 million blind children due to unavoidable cases of blindness. Better liaison of prevention of blindness program with program for control of birth defects, school health and child health care is recommended. Awareness among health care providers and the parents will improve the rehabilitation of disabled children. In countries with a high prevalence (Africa, India and China) training human resource and their placement in rural areas would be crucial. Modern technologies including telemedicine could assist eye care personnel in remote areas of countries with developing economies. However, they should be cost effective to be sustainable.
